# Endometrial biopsy performed before the first in vitro fertilization does not impact the early pregnancy rate

**DOI:** 10.1038/s41598-023-50715-y

**Published:** 2024-01-11

**Authors:** Mathilde Cellier, Sophie Werlen, Lionel Mery, Anne Genod, Bertrand Felloni, Tiphaine Semay, Béatrice Trombert, Céline Chauleur, Tiphaine Raia-Barjat

**Affiliations:** 1 Department of Gynecology and Obstetrics, Hôpital Nord, University Hospital, Avenue Albert Raimond, Saint Priest en Jarez, 42270 Saint-Étienne, France; 2 Department of Gynecology and Obstetrics, Hôpital Privé de la Loire, Saint-Étienne, France; 3 Department of Reproductive Biology, University Hospital Saint Etienne, Saint-Étienne, France; 4 Department of Public Health, University Hospital, Saint-Étienne, France; 5 Jean Monet Saint-Etienne University, INSERM, SAINBIOSE (SAnte, INgénierie, BIOlogie, Saint- Etienne) U1059, Saint-Étienne, France

**Keywords:** Health care, Medical research

## Abstract

Endometrial biopsy (EB) has been showed to increase the rate of clinical pregnancy in patients who underwent in vitro fertilization (IVF) failures. The purpose of this work was to assess the impact of an EB performed before the first in IVF on the early pregnancy rate. Be One study is a prospective, single-centre, randomized, open-label study. In this parallel study, patients were evenly split into two groups. In one group, patients underwent an EB between days 17 and 22 of the menstrual cycle that precedes the ovarian stimulation. In the other group (control), no EB was performed. The hCG-positive rate (early pregnancy rate) was evaluated on day 14 after the ovarian puncture. In total, 157 patients were randomized in the EB group and 154 patients were in the control group. The early pregnancy rate was 33.1% (52/157) in the EB group and 29.9% (46/154) in the control group (p = 0.54). Other parameters, including perforation, endometritis, or pain level were reassuring. An EB performed during the luteal phase of the menstrual cycle preceding the stimulation of the first IVF did not increase early pregnancy rate.

## Introduction

Despite the many advances made in the field of medically assisted procreation (MAP), the average success rate of in vitro fertilization (IVF) in France is approximately 24% per puncture (PP) in 2013 and 26% PP in 2014 according to the Biomedicine Agency^1^. This rate is higher and reaches a 30% success rate for pregnancies by embryo transfer (ET) as the cycles where the puncture does not result in a transfer (fertilization failure) are not taken into account. The PP pregnancy rate decreases with the number of attempts (respectively 24.2%, 21.8% and 20.5% for a 2nd, 3rd, 4th IVF)^1^.

To explain the failures, research teams have been interested in the endometrium, the tissue lining the uterine cavity, as a potentially essential factor in uterine receptivity and embryo implantation^2–7^. Previous studies, including the meta-analysis by Potdar et al*.*^8^, showed that the molecular environment could be modulated to improve the endometrial receptivity by modifying glycodelin A^9^, laminin alpha 4, integrin alpha 6 and matrix metalloproteinase 1 levels^10,11^. In particular, two manipulations have mainly been investigated to increase endometrial receptivity: a local injury of the endometrium such as an endometrial biopsy (EB) and a hysteroscopy.

Hysteroscopy (which can be combined with an EB) have been showed to result in a significant increase in pregnancy rates in patients with at least two IVF failures^12–14^. This rate significantly increased after a hysteroscopy (risk ratio (RR) 1.6 CI 95% [1.3–1.9]), with no difference between a normal examination and those finding an abnormality (RR 1 CI 95%^2^).

When only EB was performed, results were more mitigated between positive^15–19^ and no impact^20^. The biopsy would be beneficial with most effects during the month preceding IVF^21^ but could have adverse effects on the day of the embryo transfer^22^. In a meta-analysis of 6 studies, Dhulkotia et al.^23^ reported increased pregnancy rates (Odds Ratio (OR) 2.22 CI 95% [1.44–3.42]) when EB was performed in the luteal phase preceding IVF and decreased pregnancy rates during follicular puncture (OR 0.26 CI 95% [0.10–0.65]).

Three biological hypotheses have mainly been put forward to explain the favourable or unfavourable effect of EB. (1) EB could cause a local inflammatory reaction that increases uterine receptivity by promoting a cascade of biological reactions including the secretion of cytokines, growth factors, interleukins and other so-called pro-implantation proteins^24–26^. (2) EB could facilitate the synchronization between the embryo and the endometrium by correcting the advance of endometrial maturation found during the stimulated cycles^27^. Hence, the trauma caused by the biopsy would stimulate the repair processes to improve the implantation^11^. (3) EB could be responsible for an increase in the number of oestrogen receptors thus promoting endometrial maturation^28^.

The effects of endometrial manipulations have mainly been investigated in patients with at least two prior failed IVF but the impact on pregnancy rates of an endometrial manipulation before the first IVF remains unknown. Therefore, the objective of our study was to compare the early pregnancy rate, following a first IVF attempt, between patients who underwent an EB during the cycle preceding stimulation and patients who did not. EB was performed alone as it is a quick cost-effective procedure that can be easily done during a consultation by any IVF team. EB is already commonly performed in our department after IVF failures and no complications have been observed, in accordance with the literature^8,15–18^. The secondary objectives were (1) to assess the impact of an EB performed during the previous cycle stimulation before the first IVF attempt on the clinical and ongoing pregnancy rates, and on the live birth rate; (2) to describe the impact of chronic endometritis on the rate of early clinical pregnancy in patients who have undergone biopsy and (3) to assess complications related to the biopsy, in terms of pain, occurrence of acute endometritis or uterine perforation.

## Methods

### Study population

This is a prospective, single-centre, randomized, open-label study conducted in the gynaecology-obstetrics reproductive medicine department of Saint Etienne University Hospital between September 2014 and January 2018.

The study was conducted in accordance with international council for harmonisation (ICH) guidelines for good clinical practice. The protocol was approved by the south-east I personal protection committee and by the national drug safety agency (2014-A01003-044) and was registered in clinicaltrial.gouv (NCT02522806). Each patient received a leaflet explaining the study and signed an informed consent form before being able to participate in the research.

The inclusion criteria were as follows: adult patients (< 43 years old as per French regulation) preparing their first IVF attempt, having an anti Müllerian hormone (AMH) greater than 1 ng/mL, agreeing to a 2 to 12-month follow-up session and affiliated or entitled to a social security system. Patients with known intolerance to one of the proposed treatments were excluded.

### Randomization

Eligible and consenting patients were randomly assigned to two groups (ratio 1:1). In the EB group, patients underwent an endometrial biopsy between day (D) 17 and D22 of the cycle preceding ovarian stimulation. In the control group, patients did not have an endometrial biopsy. Group allocation was defined by means of sealed envelopes.

### Study protocol

Any patient consulting in the MAP department of the University Hospital of Saint-Etienne, in the context of primary or secondary infertility with a decision to IVF was informed by her referring doctor of the study. During this consultation, the study design was transparently explained, and patients were given the information leaflet, the consent form and the information sheets of the IVF protocol. After the consultation, patients were given 48–72 h to think and grant or not their free, informed, signed and revocable consent to participate in the study.

As per the standard IVF protocol, patients included in the study contacted the hospital on the first day of their period (D1). Following the usual clinical practice, patients were informed of the terms of subsequent follow-up, which include the start date of the stimulation treatment and the first monitoring consultation (ultrasound and hormonal assays). During the call, patients were also randomly split into the EB and control groups. As per study protocol, patients in the EB group were given an appointment between D17 and D22 of the cycle preceding stimulation in order to perform the EB. The stimulation protocol and follow-up of the patients were then identical in the two groups, according to the usual clinical practices.

The EB was performed using a GYNEBIOPS® pipelle. The pipelle was introduced gently through the cervix up to the uterine fundus then it was withdrawn for one centimeter. The piston was then drawn back to the end of the biopsy cannula until it self-locked, creating a negative pressure. Two or three back-and-forth movements were applied. Sample size was approximately one millimetre in diameter and five millimetres in length, and was taken from the bottom of the uterine cavity after the internal orifice of the cervix. The sample was then sent to the anatomical pathology laboratory of the University Hospital of Saint-Etienne for analysis. If endometritis was detected, patients were not excluded from the primary analysis. In case of IVF failure, patients with endometritis were given an antibiotic and anti-inflammatory treatment before proposing a new IVF. The EB procedure required no anaesthesia but could be potentially painful when the pipelle passed through the cervix. When that was the case, the pain lasted only for one to two seconds and ceased immediately after the end of the procedure.

According to our policy center, the embryo transfer took place on day two or three post-oocyte retrieval. It concerns as a priority the embryos with the highest score (Type 1 or 2) based on the morphological criteria of the classification of BLEFCO (Biologistes des Laboratoires d’Etudes de la Fécondation et de la Conservation de l’Œuf) depending on the number of cells (blastomeres), their sizes and the percentage of fragmentation. The number of embryos transferred was decided after multidisciplinary discussion between the biologist, the gynaecologist, and the couple, regardless of the study group. In the case of hight quality embryos, only one was proposed for transfer.

An absence of period between day ten and fourteen after the transfer triggered a pregnancy test by human chorionic gonadotropin (hCG) assay. A rate greater than 100 IU/ml corresponded to early pregnancy. One or two control(s) could be carried out (if 1st assay negative or > 100: stop, if 1st assay positive but < 100: 2nd assay, if > 100 or less than 1st result: stop, otherwise 3rd assay). A first endovaginal ultrasound to search for the presence of at least one intra-uterine gestational sac (IUGS) was scheduled at 5–6 weeks of gestation (WG). Then, regardless of the study group, patients benefited from the usual ultrasounds recommended during pregnancy. A negative hCG result, pregnancy termination or childbirth marked the end of the study for the patient.

Maximum study duration per patient was 12 months. Data was collected using REDCap® software.

### Evaluation criteria

The primary endpoint was the early pregnancy rate on day fourteen post-puncture, defined as a βhCG level of at least 100 IU/ml over any three assays after embryo transfer.

The secondary endpoints were as follows: the clinical pregnancy rate defined by the presence of at least one IUGS on ultrasound (US) at 5–6 WG; the ongoing pregnancy rate defined by the observation of a positive heart beat at US after 12 WG; the live birth rate; the existence of chronic endometritis defined by the pathological results of the endometrial biopsy confirming or refuting its presence; pain assessed by a visual analogue scale (VAS) requested at the end of the EB scored from 0 to 10; the occurrence of acute endometritis (spontaneous pelvic pain and/or leucorrhoea, abnormal metrorrhagia and/or hyperthermia appearing in the days following the biopsy); the occurrence of uterine perforation (intense pain at the time of the biopsy and in the days that follow).

As this was the first study on the efficacy of endometrial biopsy before the first IVF, a monitoring committee was set up to monitor patient safety. An interim analysis was carried out in the middle of the study so that this committee could rule on the safety and efficacy. We then referenced expected adverse events such as ovarian hyperstimulation syndrome and unexpected events (surgical management). We also interested in non-evolving pregnancies over the total number of patients with hCG-positive rate: biochemical pregnancy rate defined as a positive pregnancy test in the absence of any US evidence of pregnancy, and no evidence or treatment of an extra uterine pregnancy; ectopic pregnancy rate; early (< 14 WG) and late (14–22 WG) miscarriages rate defined as patients with an identified IUGS without a fetal pole or a fetal pole with no heart pulsations; fetal death in utero rate when death of a fetus occurs after 22WG; and medical termination of pregnancy rate.

### Statistical analysis

We estimated the rate of early pregnancy after first IVF within our hospital at 30% over the three years of the study (from the end of 2014 to the beginning of 2018) by analysing the data from the Saint-Etienne university hospital information systems medicalization program. This 30% rate corresponds to the positive outcome rate observed for patients who did not undergo an EB.

The meta-analysis by Potdar et al., published in 2012, grouping together studies evaluating the effect of endometrial injury, showed a relative risk (RR) of 1.71 in favour of the group with EB. By applying this RR to the rate of early pregnancy without biopsy (30%), we could expect 51% early pregnancy rate in patients with no EB. However, compared to results obtained at SEUH, all the studies included in the meta-analysis had a lower early pregnancy rate (around 25%). We therefore hypothesized that the observed effect will be lower. By considering a RR of 1.5 instead of 1.71 (i.e. an early pregnancy rate of 45% in the group with biopsy), 214 patients would have to be included per group (428 total) to reach a type-I error of 5% and a power of 90%.

The study was performed with intention to treat. The various analyses were performed using SAS V9.2 (SAS Institute) ® software. Qualitative data were analysed in terms of counts and percentages and the effect of EB was estimated with Chi2 tests. Quantitative data were described by number, mean, and standard deviation and tested depending on data normality. For normally distributed data, Student's T tests were used determine the effect of the EB. If normality was rejected, Mann–Whitney tests were performed. Regardless of the statistical test, results were considered statistically significant for type-I errors (α) of less than 5%.

## Results

### Patients’ characteristics

From September 2014 to January 2018, 315 patients were included in the study.

**Figure 1:**
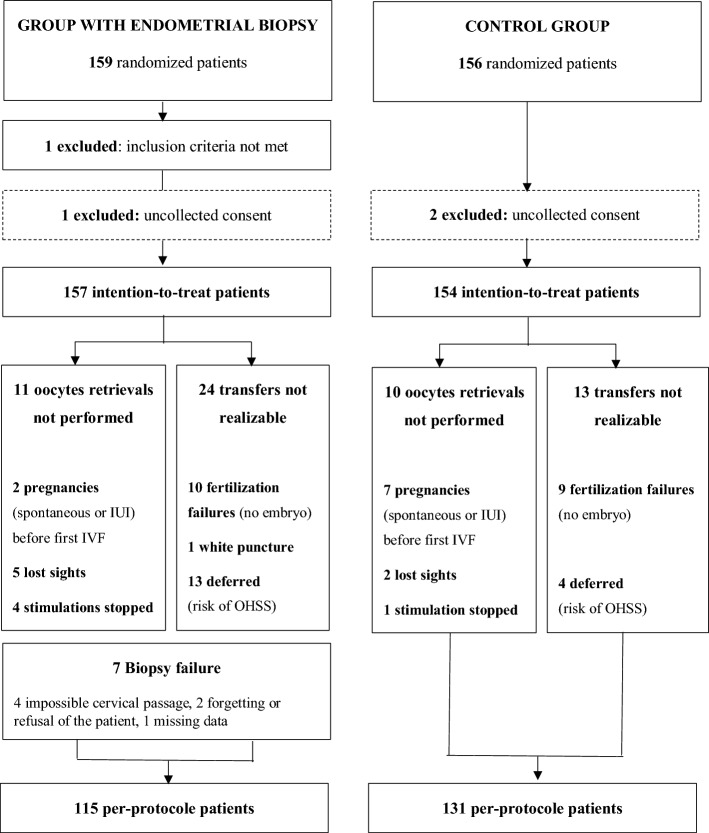
Flow chart.

A flow chart of participants is shown in Fig. 1. The intention-to-treat analysis involved 157 patients in the EB group and 154 in the control group. The endometrial biopsy completion rate in the EB group was 91% (143/157). 42 patients in the EB group (26.8%) versus 23 patients in the control group (14.9%) did not complete the entire study.

The demographic characteristics of the two groups are presented in Table 1. No significant difference was found except a higher rate of bilateral tubal obstruction in the control group and a higher sperm motility in the EB group. The groups were comparable on the type of infertility, its origin, and the associated aetiologies. The results are presented in Table 2.

**Table 1:** Demographic characteristics.

	EB group (N = 157)	Control group (N = 154)	p-value
Age (years)	31.9 ± 4.6	31.2 ± 4.6	0.191
BMI (Kg/m^2^)	24.2 ± 5.1	23.9 ± 5.2	0.692
Medical history	33 (21%)	37 (24%)	0.526
Chirurgical history	13 (8.3%)	20 (13%)	0.178
Obstetric history	65 (41.4%)	60 (39%)	0.661
Gesture	2 ± 1.4	1.6 ± 1.1	0.121
Parity	0.8 ± 0.8	0.6 ± 0.8	0.163
Early miscarriage	31 (47.7%)	26 (43.3%)	0.625
Late miscarriage	1	1	–
Fetal death in utero	0	1	–
Ectopic pregnancy (≥ 1)	9	3	–
Abortion (≥ 1)	12	17	–
Infertility			
Primary	127 (80.9%)	130 (84.4%)	0.412
Secondary	30 (19.1%)	24 (15.6%)	0.412
No of failures of IUI	1.9 ± 1.8	1.7 ± 1.8	0.350
Female infertility check up			
HSG performed	112 (71.3%)	114 (74%)	0.595
Of which abn.*	28 (25%)	23 (20.1%)	0.386
Tubal obstruction	26 (92.9%)	19 (82.6%)	0.258
Of which bilateral	9 (20%)	14 (73.7%)	0.010
HSC performed	27 (17.2%)	24 (15.6%)	0.701
Of which abn.*	15 (55.6%)	9 (37.5%)	0.197
Spermogram (WHO 2021)*			
Sperm volume (mL)	3.4 ± 1.7	3.3 ± 1.8	0.590
No of spermatozoa per mL	44.6 ± 56.8	40 ± 51.1	0.451
Motility (mean) %	43.6 ± 17.5	37.9 ± 21.3	0.011
Vitality (mean) %	62.6 ± 24.7	59.4 ± 30.2	0.316
Presence of Teratospermia**	30 (20%)	30 (20.4%)	0.930

Categorical variables reported as frequency (percentage) and continuous variables reported as mean ± standard deviation. Abn, abnormality; BMI, body mass index; HSG, hysterosalpingography; HSC, hysteroscopy; IUI, intra uterine insemination.

*Calculation of values performed without missing values.

**David modified classification.

**Table 2:** Demographics—type of infertility.

	Primary infertility			Secondary infertility		
	EB group N = 127	Control group N = 130	p-value	EB group N = 30	Control group N = 24	p-value
Female causes*	63 (49.6%)	58 (44.6%)	0.423	9 (30%)	9 (37.5%)	0.561
Tubal, uterine, or cervical abnormalities	35	22		8	6	
Pelvic adhesions	8	5		2	0	
Ovulation abnormality	29	32		4	3	
Of which PCOS	24	24		3	2	
Endometriosis	9	8		3	0	
Cervical hostility	1	4		1	0	
Genetics (Turner syndrome, translocation 2–10)	0	1		0	0	
Male causes* (spermogram abnormalities)	56 (44.1%)	65 (50%)	0.343	12 (40%)	12 (50%)	0.462
Unexplained causes	30 (23.6%)	32 (24.6%)	0.852	9 (30%)	6 (25%)	0.684

*Possible mixed causes intricate.

In the EB group, 122 (77.7%) patients benefited from an embryo transfer against 131 (85%) in the control group. The reasons for non-transfer are detailed in the flow-chart (Fig. 1) and include oocyte retrievals not performed and transfers not realizable. The Table 3 presents the different types of embryos transferred within the two groups. No statistical difference was observed at this level neither in quantity (number of embryos transferred) nor in quality (similar type of embryos transferred).

**Table 3:** Type* of embryos transferred.

	EB group (N = 122)		Control group (N = 131)		p-value
Number of embryos transferred	1 53 (43%)	2 69 (57%)	1 54 (41%)	2^and more^ (**) 77 (59%)	0.721
Single embryo transferred	N = 53		N = 54		0.470
T1 Type	41 (77.4%)		43 (79.6%)		
T2 Type	11 (20.7%)		8 (14.8%)		
≥ T3 Types	1 (1.9%)		3 (5.6%)		
Two embryos transferred		N = 69		N = 77	0.243
Type 1 + Type 1		15 (21.7%)		12 (15.6%)	
Type 1 + Type 2		15 (21.7%)		15 (19.5%)	
Type 1 + Type ≥ 3		1 (1.5%)		9 (11.7%)	
Type 2 + Type 2		18 (26.1%)		17 (22%)	
Type 2 + Type ≥ 3		10 (14.5%)		12 (15.6%)	
Type ≥ 3 + Type ≥ 3		10 (14.5%)		12 (15.6%) **	

*Daily observation of embryos from the 2nd day of development focused on different morphological criteria (of good or low quality): the number of blastomeres, the typical character or not of these blastomeres (shape, relative volume, arrangement), the rate of cytoplasmic fragmentation (< 10%, 10–30%, 30–50%, > 50% of the embryonic volume), and the possible presence of multinucleated blastomeres (BMN).

The embryos were then divided into 5 types, according to decreasing embryonic quality, types 1 and 2 being considered as “top embryos” (carrying at most one low quality criterion), according to the same criteria as the BLEFCO classification.

**Only one patient benefited from the transfer of 3 embryos (3 ≥ Type 3) and was included here.

### Relationship between EB realized before the first IVF on the hCG-positive rate

The rate of hCG-positive was 33.1% (52/157) in the EB group and 29.9% (46/154) in the control group with no difference in the two groups in intention to treat (p = 0.54) (Table 4).

**Table 4:** Early pregnancy rate, clinical pregnancy rate, ongoing pregnancy rate and live birth rate.

	EB group (N = 157)	Control group (N = 154)	p-value
Early pregnancy rate (B-HCG > 100 mlU/ml)	52 (33.1%)	46 (29.9%)	0.537
B-HCG > 100 (1rst test)	23 (14.6%)	28 (18.2%)	
B-HCG > 100 (2nd test)	21 (13.4%)	13 (8.4%)	
B-HCG > 100 (3rd test)	8 (5.1%)	5 (3.2%)	
Clinical pregnancy rate (Presence of at least one intra uterine gestational sac at 6 WG)	45 (28.7%)	45 (29.2%)	0.914
1 IUGS	39	39	
2 IUGS	6	6	
Ongoing pregnancy (Presence of ongoing pregnancy at 12 WG)	38 (24.2%)	41 (26.6%)	0.624
Live birth	37 (23.6%)	40 (26%)	0.623

### Relationship between EB realized before the first IVF on the clinical pregnancy rate, ongoing pregnancy rate, and live births

Clinical pregnancy, ongoing pregnancy, and live births rates were similar between the two groups (Table 4). Gestational age at delivery for live births was 38.5 WG (± 2.8 WG) in the EB group versus 38.7 WG (± 1.8 WG) in the control group (p = 0.73). There were as many occurrences of twin pregnancies in both groups.

### Relationship between EB realized before the first IVF on pregnancy outcomes (excluding live birth)

Pregnancy outcomes, excluding live births, in patients with an initial positive pregnancy test are presented in Table 5. When taken individually, pregnancy outcomes between the two groups were not found statistically different. However, when biochemical pregnancy rates and early miscarriage number were combined, a significant difference was observed with 25.9% of patients (15/52) in the EB group presenting a poor outcome compared to 10.4% of patients (5/46) in the control group (p = 0.043).

**Table 5:** Pregnancy outcomes (excluding live birth) in patients who presented a positive biological pregnancy test (B HCG > 100 mIU/ml) on D14.

	Group EB (N = 52)	Control group(N = 46)	p-value
Biochemical pregnancy	5	2	–
Early miscarriage	10	3	p = 0.064
Late miscarriage	0	1	–
Fetal death In utero	0	1	–
Ectopic pregnancy	2	0	–
Medical termination < 22 WG	2	1	–

### Relationship between absence or presence of chronic endometritis and the occurrence of early pregnancy rate

In the EB group, 30 chronic endometritis (or doubtful results) were detected, including 9 patients (30%) who presented a hCG-positive result. Statistical analysis did not retain a link between the absence or presence of chronic endometritis and the occurrence of hCG-positive (p = 0.69).

### Relationship between EB realized before the first IVF and the occurrence of complications

In our study, no complication related to the endometrial biopsy performed during the luteal phase preceding IVF was reported. In particular no occurrence of acute endometritis or uterine perforation was observed.

The evaluation of the pain of the EB was carried out using a VAS of the pain. The pain was estimated on average at 3.02 (± 1.77) with very variable ratings for each of the patients ranging from 0 to 9 (for a minimum set at 0 and a maximum at 10).

Ovarian hyper stimulation concerned 13.3% of patients in the EB group against 8.3% in the control group (p = 0.163). This syndrome was in most cases described around the transfer date. Two patients in the EB group had to be surgically managed around 10 AW in a context of adnexal torsions related to the observed hyper stimulation. Two patients in the EB group presented an ectopic pregnancy. The first was medically managed and the second of cornual localization, had to be surgically operated by laparotomy for hemoperitoneum.

## Discussion

Performing an endometrial biopsy, during the luteal phase of the cycle preceding the stimulation of a first IVF, did not increase early pregnancy rate and could be associated with an increase in early spontaneous miscarriages.

The impact of EB has recently attracted a lot of attention with multiple studies being carried out in parallel to ours^29–33^ and complementing prior work^17–19^. The average VAS pain score in our study was 3 in accordance with the 2012 Cochrane who reported a pain score of 4.6^34^. Although the statistical power required for the study was not reached, the results were in line with the recent literature^35,36^. Live birth in patients with endometrial manipulation before IVF (versus controls) were reported at 26% in both group in a study carried out in 2019^29^ and at 23.7% (versus 19.1%) in a study carried out in 2021 (Scratch study^30^). In 2019, Farquhar et al.^31^ recommended to stop endometrial scratching based on the work of Lensen et al.^29^ and a meta-analysis^32^ which reported no statistically different outcomes in patients who underwent endometrial manipulation compared to controls.

Nevertheless, this is a subject that is still under scrutiny on the weather or not it should be performed. Some recent studies from this year have favourable outcome toward scratching^37,38^. Moreover, the meta-analysis by Van Hoogenhuijze et al. in 2019^33^ suggests a beneficial effect of EB mainly in a subgroup of the infertile population: the impact of the biopsy would be more pronounced in patients with repeated implantation failures who underwent at least than two IVFs^34,39,40^. Coughlan et al.^41^ define this implantation failure as the absence of progress of a clinical pregnancy after transfer of at least four good quality embryos in a minimum of three fresh or frozen transfers in a woman under 40 years old.

Today, EB needs to be put back into context with the raise of Omics techniques which attracted a lot of attention over the past decade in the field of fertility. Omics are currently considered as innovative and primordial biomarkers in the assessment of endometrial receptivity. The advent of endometrial receptivity assessment tests encouraged practitioners to revisit their practices. This led some laboratories to push further and to offer EB as a predictive treatment. A French laboratory (MatriceLAB Innove)^42–44^ proposes to draw up a profile of uterine receptivity during the implantation window by analyzing endometrial immunological markers (on a prior EB), in patients with IVF failure. The aim is to understand the mechanisms at the origin of these repeated failures and to be able to propose an appropriate therapy. The underlying mechanisms, whether based on an immunological rebalancing, or a response to an aggression, that would explain increases in pregnancy rates remain unknown. In our study, no link was observed between the rate of early pregnancy and the presence of an inflammatory state caused by a chronic endometritis (p = 0.69).

But which entity, between aggression and immunological rebalancing, is ultimately beneficial to the pregnancy rate? Ledee et al.^7^ investigated the impact of uNK cells in the uterine stroma. In the peri-implantation period, 50–70% of the stroma is composed of uNK cells (CD56 bright) and this rate increases throughout pregnancy. uNK cells are essential for the invasion, differentiation, and normal growth of the trophoblast. They are also involved in the modulation of the Mother-Concept dialogue and neoangiogenesis. The absence or immaturity of uNK cells is responsible for apposition problems, abnormal angiogenesis, and defects in the establishment of conceptus growth mechanisms. On the contrary, an excess or hyperactivation of these cells is responsible for problems of invasion, disturbance of immunotolerance as well as generalized local apoptosis. The counting of uNK cells on EB in the luteal phase (MatriceLAB) made it possible to draw up distinct immunological profiles and consequently to propose more specific treatments to increase the pregnancy rate^45,46^. These investigations on patients with repeated implantation failures seem to be promising but only for a given population of women. However, recents publications from 2021 onwards progressively show that such tests have very limited capacity of improving clinical outcomes and in some cases might present poorer outcomes^47^. In addition, the use of this biopsy has a high cost and is currently not reimbursed, while one of the major advantages of EB before IVF was to improve the pregnancy rate in a simple and economical way.

Moreover, according to the Be-One data, the realization of an EB, during the luteal phase of the cycle preceding the stimulation of a first IVF, could be associated with an increase in early spontaneous miscarriages when this is associated with the biochemical pregnancy rate (25.9% with EB versus 10,4% without). This result is inconsistent with previous work. In the Cochrane review by Lensen et al. in 2021 which was based on 38 trials, endometrial lesions were not linked to miscarriage rates^48^. The same conclusions were drawn for hysteroscopies performed before IVF^49^.

Compared to EB, hysteroscopy enable the diagnosis and treatment of endocavitary pathologies that may be responsible for a decline in fertility. The discovery of an endocavitary polyp or a submucosal myoma during an infertility assessment requires their hysteroscopic resection in view of the positive impact on the subsequent pregnancy rate^50^. Approximately 12% of hysteroscopies can reveal an unsuspected abnormality during the initial assessment^51^. In patients under GnRH agonists, ambulatory hysteroscopies associated with an endometrial biopsy made it possible to diagnose abnormalities in 23% of patients (incomplete septum, chronic endometritis, polyp and endometrial hyperplasia)^52^. Moreover, the pregnancy rates after hysteroscopy were significantly higher compared to the control group (31% vs 21%, p = 0.0035) with identical early miscarriage rates in the two groups (19.4 vs 19%). Finally, a meta-analysis of 10 studies showed that hysteroscopy performed before IVF attempts significantly improved clinical pregnancy and delivery rates (p < 0.00001). However, the study by El Khayat et al.^53^ does not show any significant difference in the clinical pregnancy rate when performing EB in combination with hysteroscopy compared to hysteroscopy alone.

## Conclusion

The randomized clinical trial Be One show that performing an endometrial biopsy, during the luteal phase of the cycle preceding the stimulation of a first IVF, did not increase early pregnancy rate and could be associated with an increase in early spontaneous miscarriages.

We therefore recommend stopping the practice of endometrial scratching before the first IVF which is based only on empirical data. This result could however be refined if the knowledge of the receptivity of the endometrium was known, in particular for patients after repeated failures of implantation. The metagenomic analysis of the endometrial microbiome, the immunohistochemical characterization of stromal cells, or the immune study of natural killer cells, could lead to more specific indications related to the scratching of the endometrium depending on the types of infertility.

## Data Availability

The datasets generated during and/or analysed during the current study are available from the corresponding author on reasonable request.
