# Correction: Endometrial biopsy performed before the first in vitro fertilization does not impact the early pregnancy rate

**DOI:** 10.1038/s41598-026-44043-0

**Published:** 2026-03-17

**Authors:** Mathilde Cellier, Sophie Werlen, Lionel Mery, Anne Genod, Bertrand Felloni, Tiphaine Semay, Béatrice Trombert, Céline Chauleur, Tiphaine Raia-Barjat

**Affiliations:** 1 Department of Gynecology and Obstetrics, Hôpital Nord, University Hospital, Avenue Albert Raimond, Saint Priest en Jarez, 42270 Saint-Étienne, France; 2 Department of Gynecology and Obstetrics, Hôpital Privé de la Loire, Saint-Étienne, France; 3 Department of Reproductive Biology, University Hospital Saint Etienne, Saint-Étienne, France; 4 Department of Public Health, University Hospital, Saint-Étienne, France; 5 Jean Monet Saint-Etienne University, INSERM, SAINBIOSE (SAnte, INgénierie, BIOlogie, Saint- Etienne) U1059, Saint-Étienne, France

Correction to: *Scientific Reports* 10.1038/s41598-023-50715-y, published online 11 January 2024

The original version of this Article contained an error in the name of the author Lionel Mery, which was incorrectly given as Mery Lionel.

The original Article has been corrected.

